# The effects of nail rigidity on fracture healing in rats with osteoporosis

**DOI:** 10.1080/17453670902807490

**Published:** 2009-02-01

**Authors:** Mo Sha, Zheng Guo, Jun Fu, Jing Li, Chao Fan Yuan, Lei Shi, Shu Jun Li

**Affiliations:** ^1^Department of Orthopedics, Xijing Hospital, Fourth Military Medical UniversityXi'anChina; ^2^Shenyang National Laboratory for Materials Science, Institute of Metal Research, Chinese Academy of SciencesShenyangChina

## Abstract

**Background and purpose** Stress shielding from rigid internal fixation may lead to refracture after removal of the osteosynthesis material. We investigated the effect of a low-rigidity (Ti-24Nb-4Zr-7.9Sn) intramedullary nail regarding stress shielding and bone healing of osteoporotic fractures in the rat.

**Methods** 40 female Sprague-Dawley rats, aged 3 months, were divided into the following groups: sham-operation (SHAM) (n = 10), ovariectomized (OVX) (n = 10) and OVX-fracture (n = 20). 10 SHAM rats and 10 OVX rats were killed after 12 weeks to provide biomechanical data. Ovariectomy was performed 12 weeks before fracturing both femurs in 20 rats. The left fracture was stabilized with a high-rigidity titanium alloy pin (Ti-6Al-4V; elastic modulus 110 GPa) and the right with a low-rigidity (Ti-24Nb-4Zr-7.9Sn; elastic modulus 33 GPa). The bony calluses were examined by micro-CT at 6 and 12 weeks after fracture, bone volume (BV) and total volume (TV) were determined at the callus region (ROI1) and the total femur (ROI2). Subsequently, the bones were tested mechanically by a three-point bending test.

**Results** In the low-rigidity group, TV (ROI1) increased at 6 weeks, but BV (ROI1), BV (ROI2) were similar but maximum load increased. At 12 weeks, the maximum load and also BV (ROI1, ROI2) were increased in the low-rigidity group.

**Interpretation** The low-rigidity nail manufactured from Ti-24Nb-4Zr-7.9Sn showed better external callus formation, seemed to reduce effects of stress shielding, and reduced bone resorption better than the stiffer nail. The low-rigidity nail was strong enough to maintain alignment of the fracture in the osteoporotic rat model without delayed union.

## Introduction

Stress shielding because of rigid internal fracture fixation may weaken the bone; a substantial number of refractures have been reported after removal of rigid internal fixators (Deluca et al. 1988). Several in vivo studies have shown stress shielding from the use of rigid internal fixation (Terjesen 1984, Terjesen and Apalset 1988). Less rigid fixators may improve fracture healing and prevent bone weakening due to stress shielding (Amnon al. 1997, Fujihara et al. 2003).

In osteoporotic fractures, the effects of stress shielding will be more obvious because of the lower rigidity of the bone, with longer healing time requiring longer time of fixation (Comekoglou et al. 2007). However, the effect of nail rigidity on bone healing of osteoporotic fractures has hardly been investigated (Ryan 1991).

Flexible fixators have not gained acceptance in trauma care, as the material strength to maintain fragment apposition and alignment of the bone has been in question (Terjesen and Johnsson 1986).

The combination of good mechanical properties and biochemical compatibility makes titanium alloys a suitable material for orthopedic implants. However, the elastic modulus of bio-titanium alloys (Ti-6Al-4V,110GPa) currently in use is higher compared with that of human bone (10–20 GPa), which may lead to stress shielding, especially in osteoporotic fractures.

Ti-24Nb-4Zr-7.9Sn (wt %) is a new β-type titanium alloy that has been developed for biomedical applications (Hao et al. 2005, 2007). Ageing treatment in the (a + β) two-phase field increases both strength and Young’s modulus, and results in a combination of high strength (800–900 MPa), better than Ti-6Al-4V (600 MPa), and relatively low elastic modulus (33 GPa). Theoretically, this means that fixators manufactured from this new β-type titanium alloy may have enough strength to maintain fragment apposition and alignment but may have reduced stress shielding.

We evaluated the effects of high-rigidity and low-rigidity nails (made of Ti–24Nb–4Zr–7.9Sn or Ti-6Al-4V) on mineralization and strength of callus in osteoporotic femoral fractures in rats.

## Materials and methods

### Animal surgery and care

This investigation followed international guidelines for protection of animals and was approved by the ethics committee of the Department of Surgery at our institution.

40 female Sprague-Dawley rats, 3 months old and weighing 241 ± 16 g, were acclimatized for 1 week in our laboratory before the experiments. They were exposed to a cycle of 12 h light and 12 h dark at 22°C. The animals had free access to standard laboratory chow and water ad libitum.

The rats were divided into the following 3 groups: SHAM (sham-operation) rats (n = 10), OVX (ovariectomized) rats (n = 10), and OVX-fracture rats (n = 20). Following intraperitoneal anesthesia (pentobarbital at 5 mg/100 g body weight), a ventral incision was made in all 40 rats and the ovaries were removed from 30 after ligation of the uterine horns. The other 10 rats thus underwent a sham operation (only incision) under the same anesthesia. 3 months after the OVX procedure, all animals were assessed for total body bone mineral density using dual-energy X-ray absorptiometry (DEXA Lunar; GE Healthcare) to confirm the usefulness of the OVX group as an osteoporosis model. If the OVX animals had statistically significantly lower bone mineral density than the SHAM animals at this time, the fracture procedure was initiated; otherwise, it was postponed until 4 months. 10 SHAM rats and 10 osteoporotic rats were killed at the end of the 12-week experiment to provide biomechanical data. With the remaining 20 animals, a standardized surgical procedure was performed (Utvag et al. 2001): following intraperitoneal anesthesia, the left femur was exposed between the lateral vastus and hamstrings. The muscles were elevated laterally in part and a partial osteotomy involving one-third of the diaphysis was made with a fine-toothed circular saw blade mounted on an electrical drill. The osteotomy was done at the end of the trochanteric ridge approximately 13 mm from the top of the greater trochanter, and the femur was then broken manually. The medullary canal was successively reamed from the osteotomy site in the proximal direction through the top of the greater trochanter and in the distal direction to the level of the condyles to a diameter of 1.6 mm, using steel burrs mounted on the electric drill. This procedure secured a central position of the reamer in the medullary cavity. The fracture was manually reduced, and a nail was inserted from the trochanter to the femoral condyles for stabilization. The right femurs were given the same operation. Two types of nail (Shenyang National Laboratory for Materials Science, Institute of Metal Research, Chinese Academy of Sciences) were used in the experiment. The left femur received a high-rigidity titanium nail (Ti-6Al-4V; elastic modulus 110 GPa with a bending rigidity of 272 (SD7.1) N/mm; maximum load: 172 (SD 5.2) N) whereas the right femur received a low-rigidity titanium nail (Ti-24Nb-4Zr-7.9Sn; elastic modulus 33 GPa with a bending rigidity of 81 (SD 5.2) N/mm; maximum load: 265 (SD 8.2) N). The rigidity and maximum load of the nails were determined in vitro.

All nails were cylindrical, with a diameter of 1.6 mm and a length of 36 mm, and were rubbed with 1,000-grit silicone paper to produce a uniform surface roughness. Fixation was achieved without radiographic control. Proper nail placement was confirmed with radiographs taken at the end of the experiment. The wounds were closed in two layers.

### Specimens

Anesthetized animals were killed at 6 and 12 weeks after fracture using an overdose of sodium pentobarbital (10 mg/kg) delivered by cardiac puncture. Both femurs were harvested and stripped of overlying skin and muscle tissue. The bones were stored in sealed test tubes at –80°C.

### Micro-CT imaging

The bones were examined by micro-CT after removal of the nails. They were scanned using a GE Explore Locus SP Pre-Clinical Specimen Micro-CT (GE Medical Systems) operated at a 13-µm isotropic voxel resolution. Bone tissue was separated from non-bony tissue using the thresholding algorithm provided by the manufacturer of the Micro-CT unit. The output density data (Hounsfield units) was converted to mineral content (g/cm^3^) using the density data from the phantoms. A 5-mm region of interest (ROI1) between 11 and 16 mm from the top of the greater trochanter, corresponding to the callus region, and total femur (ROI2) was measured. Bone volume (BV) and total volume (TV) were calculated.

### Mechanical testing

The specimens were placed in a moisture chamber at room temperature prior to mechanical testing. A 3-point bending test was performed, applying bending in the plane of natural extension. The bones were placed in a jig with 2 horizontal bars (each 3 mm in diameter) mounted on roller bearings. The distance between the axes of the bars was 13 mm. The midpoint was a blunt metal edge moved by the crosshead of a mechanical testing machine (MTS 880; MTS Systems Corp., Eden Prairie, MN). The femur was mounted with the lesser trochanter proximal to and in contact with the proximal transverse bar of the jig. This arrangement placed the point of application of force over the fracture site at the end of the trochanteric ridge, which corresponds to the narrowest part of the medullary cavity. However, when the femur had angular or rotational deformities, approximation of this arrangement was made and the bone was placed in a stable position to avoid slippage during testing. Bending was applied successively as determined by the degree of strain (ductility) of the femur by the Test Star software programme (Utvag, Tromsoe, Norway). The maximum bending load at failure (N) was interpreted as the strength of the bones.

### Statistics

Data were expressed as mean (SD) and analyzed using paired t–test. Significance was set at p-values of < 0.05. Statistical analysis was performed using SPSS version 11.0 software.

## Results

### Micro-CT and clinical results (Table and Figure 1)

1 femur broke during the operation, and 1 rat died postoperatively. The other animals tolerated the operation well and resumed running on the first postoperative day. 1 animal was excluded due to proximal migration of the nail after 6 weeks.

**Figure 1.**
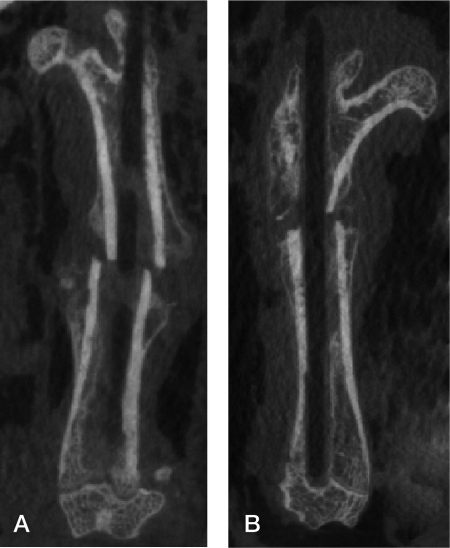
Coronal micro-CT scan at 6 weeks. A. Low rigidity group (Ti-24Nb-4Zr-7.9Sn). B. High rigidity group (Ti-6Al-4V). The intramedullary devices have been removed for reduction of metal artifact.

**Figure 2.**
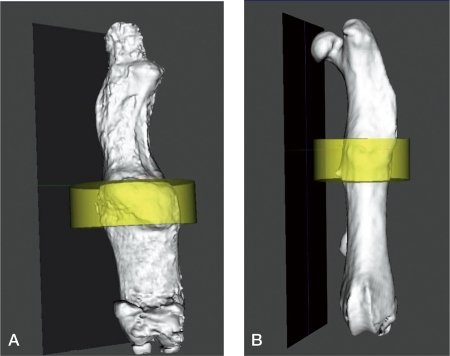
Three-dimensional reconstruction at 12 weeks (with yellow region corresponding to ROI1). A. Low-rigidity group. B- High-rigidity group.

**Table:** Bone volume (BV) and total volume (TV) in 2 regions of interest (ROI1 and ROI2), and maximum load at 6 and 12 weeks. Values represent mean (SD)

	BV (ROI1) (mm^3^)	TV (ROI1) (mm^3^)	BV (ROI2) (mm^3^)	Maximum load (N)
*6 weeks (n = 8)*				
Low-rigidity	75 (12)	819 (92)	327 (26)	36 (7)
High-rigidity	70 (6)	553 (40)	328 (24)	45 (9)
Difference	4 (15)	266 (72)	-2 (42)	-9 (13)
95% CI	-8–17	206–326	-37–33	-20–2
Test statistic	0.86	10	-0.11	-1.0
p-value	0.4	< 0.001	0.9	0.09
*12 weeks (n = 9)*				
Low-rigidity	90 (9)	437 (35)	338 (23)	125 (20)
High-rigidity	78 (9)	401 (33)	303 (22)	109 (15)
Difference	12 (10)	36 (32)	34 (25)	17 (19)
95% CI	5–21	12–61	15–53	2–31
Test statistic	3.7	3.4	4.2	2.6
p-value	0.006	0.01	0.003	0.03

The fractures healed by the production of external callus. Micro-CT revealed clearly visible fractures at 6 weeks, but they were scarcely visible at 12 weeks.

Micro-CT at ROI1 showed different mechanical conditions at the site of osteotomy for the different nails. At 6 weeks, the mean fibrous callus area (TV) of the low-rigidity fixator group was higher than in the high-rigidity group (low-rigidity: 819 mm^3^; high-rigidity: 553 mm^3^; p < 0.001), and it remained higher at the 12-week examination (low-rigidity: 437 mm^3^; high-rigidity: 400 mm^3^; p = 0.01).

The mineralized bone volume (BV) was similar in both groups at 6 weeks but increased considerably after that in the low-rigidity fixator group over the next 6 weeks (low-rigidity: 90 mm^3^; high-rigidity: 78 mm^3^; p = 0.006).

ROI2 showed different effects of stress shielding. The BV of ROI2 was similar in both groups at 6 weeks but increased in the low-rigidity group at 12 weeks (low-rigidity: 337 mm^3^; high-rigidity: 303 mm^3^; p = 0.003).

At 12 weeks, the femur in the low-rigidity group was stronger than in the high-rigidity group (Figures 3 and 4).

### Mechanical testing

The bending rigidity and maximum load of the intact femurs in OXV rats were 86 (SD 24) N/mm and 176 (SD34) N, respectively. In the SHAM rats, the corresponding figures were 244 (SD38) N/mm and 372 (SD43) N.

Maximum load was similar in both groups at 6 weeks, but at 12 weeks it was increased in the low-rigidity group (low-rigidity: 125 (SD 20) N; high-rigidity: 109 (SD 15) N; p = 0.03).

## Discussion

Clinical and experimental studies have shown that rigid internal fixators impair fracture healing, increase cortical resorption, and result in mechanical weakening of the fractured bone, which will be more obvious in osteoporotic fractures (Davidson et al. 2001). Several authors have demonstrated that low-rigidity fixation promotes fracture healing and strengthens the callus (Terjesen 1984, Terjesen and Johnssson 1986, Terjesen and Apalset 1988, Ryan 1991, Utvag and Rekerås 1998, Utvag et al. 2001). However, it is unclear whether low-rigidity internal fixators provide sufficient stability to maintain fracture alignment. There may also be a risk that reduced rigidity would lead to increased interfragmentary motion and prolonged healing, especially in osteoporotic fractures. Terjesen and Apalset (1988) found that flexible plates, made of stainless steel or glass fiber-reinforced epoxy, increased the risk of redislocation and mechanical failure.

Wang et al. (1985) concluded that the optimum rigidity for an intramedullary femoral nail to stimulate external callus formation and bone healing was between 20% and 50% of femoral bending rigidity; insufficient rigidity produced unreliable and widely variable bone healing. However, Wang’s conclusion has not gained wide acceptance in the clinic, and it remains unclear which biomechanical conditions are optimal for bone healing in osteoporotic fractures (Zaki et al. 1998).

With advancements in new materials with improved mechanical characteristics, nails with lower rigidity but sufficient mechanical strength have been introduced. Ti–24Nb–4Zr–7.9Sn, a new β-type titanium alloy, is particularly useful in biomedical applications, with a good mechanical compromise between a high degree of hardness (800–900 MPa) and low Young’s modulus, making it well suited for use in bone implants. The elasticity of Ti-24Nb-4Zr-7.9Sn matches that of bone (33 GPa) more closely than other implant materials.

We found that the optimum rigidity for an intramedullary femoral nail to stimulate external callus formation and bone healing was 30% of normal femoral bending rigidity (90% of the osteoporotic femoral bending rigidity). The high-rigidity nails (with 300% of the bending rigidity of osteoporotic femur) reduced bone strength. Furthermore, the low-rigidity nails reduced the effects of stress shielding and resulted in less bone resorption than the stiffer nails, but were strong enough to provide sufficient stability to maintain alignment and position for the femoral shaft fractures in our osteoporotic rat model.

The ovariectomized rat is the animal model most studied for osteoporosis. However, in its guidelines for preclinical and clinical evaluation of agents used in the treatment or prevention of postmenopausal osteoporosis, the US Food and Drug Administration (FDA) recommends that agents be evaluated in 2 different animal species, including ovariectomized rats and a second, non-rodent large animal model that has Haversian systems and remodeling patterns similar to the human situation (FDA 1994). The lack of the Haversian system in cortical bone, and the absence of multicellular unit-based remodeling in young rats limits the usefulness of our study (Egermann et al. 2005). Additional comparative studies will be necessary to examine the effects of nail rigidity on bone healing of non-rodent large animal models with osteoporosis.
